# Musical Mnemonics in Cognitively Unimpaired Individuals and Individuals with Alzheimer’s Dementia: A Systematic Review

**DOI:** 10.1007/s11065-023-09585-4

**Published:** 2023-04-14

**Authors:** Marije W. Derks-Dijkman, Rebecca S. Schaefer, Roy P. C. Kessels

**Affiliations:** 1 Donders Institute for Brain, Cognition and Behaviour, Neuropsychology & Rehabilitation Psychology, Radboud University, PO Box 9104, 6500 HE Nijmegen, The Netherlands; 2 Health, Medical & Neuropsychology Unit, Institute for Psychology, Leiden University, Leiden, The Netherlands; 3 Leiden Institute for Brain and Cognition, Leiden University, Leiden, The Netherlands; 4 Academy of Creative and Performing Arts, Leiden University, Leiden, The Netherlands; 5 Centre of Excellence for Korsakoff and Alcohol-Related Cognitive Disorders, Vincent Van Gogh Institute for Psychiatry, Venray, The Netherlands; 6 Department of Medical Psychology & Radboud Alzheimer Center, Radboud University Medical Center, Nijmegen, The Netherlands

**Keywords:** Musical mnemonics, Working memory, Episodic memory, Aging, Alzheimer’s dementia, Musical expertise

## Abstract

Based on the idea that music acts as a mnemonic aid, musical mnemonics (i.e., sung presentation of information, also referred to as ‘music as a structural prompt’), are being used in educational and therapeutic settings. However, evidence in general and patient populations is still scarce. We investigated whether musical mnemonics affect working and episodic memory performance in cognitively unimpaired individuals and persons with Alzheimer’s dementia (AD). Furthermore, we examined the possible contribution of musical expertise. We comprehensively searched the PubMed and PsycINFO databases for studies published between 1970 and 2022. Also, reference lists of all identified papers were manually extracted to identify additional articles. Of 1,126 records identified, 37 were eligible and included. Beneficial effects of musical mnemonics on some aspect of memory performance were reported in 28 of 37 studies, including nine on AD. Nine studies found no beneficial effect. Familiarity contributed positively to this beneficial effect in cognitively unimpaired adults, but require more extensive investigation in AD. Musical expertise generally did not lead to additional benefits for cognitively unimpaired participants, but may benefit people with AD. Musical mnemonics may help to learn and remember verbal information in cognitively unimpaired individuals and individuals with memory impairment. Here, we provide a theoretical model of the possible underlying mechanisms of musical mnemonics, building on previous frameworks. We also discuss the implications for designing music-based mnemonics.

**Supplementary Information:**

The online version contains supplementary material available at 10.1007/s11065-023-09585-4.

## Introduction

There is a popular and long-held belief that music can serve as a mnemonic device by setting information that has to be learned and remembered to music (Moussard et al., 2012; Rainey & Larsen, 2002). The strong statement of Sloboda (1985; p. 268) that ‘music is of immense benefit as a mnemonic aid’ has been adopted by various authors (Rainey & Larsen, 2002; Silverman, 2010), and the use of music for the facilitation of memory performance has been called ‘music as a structural prompt’ (Madsen et al., 1975). In educational and therapeutic settings, music has often been paired with social and academic skills to be learned (e.g., Jellison, 1976; Jellison & Miller, 1982; Ludke et al., 2014; Wolfe & Hom, 1993). In primary school, children learn the ABC-song, whereby the alphabet is sung to a familiar melody (i.e., ‘Twinkle, twinkle, little star’), to support the acquisition and recall of letters and their proper order in the alphabet (Jellison, 1976; Wolfe & Hom, 1993). Furthermore, children learn for example to identify body parts by singing the lyrics “Head, shoulders, knees and toes” (Wolfe & Hom, 1993). Others have offered a fun and innovative approach to learning physics through the use of karaoke (Dickson & Grant, 2003). Moussard et al. (2012) asserted that music is also used for other purposes related to memory and association, for example in advertisements on television (e.g., Yalch, 1991). Moreover, more general claims about the effects of music listening and cognitive performance are widespread (e.g., Schellenberg & Weiss, 2013, see also Box 1 on the ‘Mozart Effect’). Empirical evidence on the beneficial effects of using musical mnemonics is, however, limited (Rainey & Larsen, 2002), and studies so far have largely been conducted in cognitively unimpaired individuals (Moussard et al., 2012).

Box 1 Music and Its Influence on Cognition The number of publications on the assumed positive effects of music on cognitive functioning has increased considerably after the publication of Rauscher and colleagues (1993), presenting the ‘Mozart Effect’. After listening to Mozart’s piano sonata K448, the researchers observed a brief improvement in reasoning skills solving spatial problems in cognitively unimpaired individuals. Although the specificity of Mozart’s music was subsequently invalidated, and the finding identified as an effect of mood and arousal on cognition (Thompson et al., 2001), various studies evaluating this contextual effect of music (i.e., mere listening) on cognition have been carried out, also using other cognitive tasks, and music of other composers. For example, Mammarella et al. (2007) showed better working memory performance in cognitively unimpaired older adults on digit span tasks after listening to Vivaldi. The mood-arousal hypothesis is also supported by neuroimaging evidence. In an overview of studies using positron emission tomography (PET) and functional magnetic resonance imaging (fMRI), Pauwels et al. (2014) argued that listening to pleasurable music, due to evoked emotions, gives higher arousal (among other things in the amygdala and hippocampus and the orbitofrontal cortex, para-hippocampal gyrus and temporal lobes) resulting in temporarily enhanced cognitive performance in multiple domains. While the specific ‘Mozart Effect’ is now generally considered a neuromyth (MacDonald et al., 2017), it appears that listening to music can indeed affect cognition through arousal mechanisms. However, these studies focused on the contextual (transfer) effects of listening to music before the performance of a cognitive task, which is not the same as the use of musical mnemonics, and thus beyond the scope of this review.

### Musical Mnemonics: A Possible Tool for Cognitive Rehabilitation in Memory-Impaired Individuals?

The question whether musical mnemonics may have clinical relevance for memory rehabilitation was posed by Moussard et al. (2012) in their case study in a person with mild AD. They reviewed the two only existing studies at the time in participants with memory impairments due to AD (Prickett & Moore, 1991; Simmons-Stern et al., 2010) and showed an advantage of a sung presentation in persons with AD despite methodological or task-specific issues. Simmons-Stern et al. (2010), for example, referred to an anecdote in which the daughter of a person with AD successfully taught her non-musician father about current events through singing the new stories to the melody of a popular song, suggesting that AD non-musicians may also benefit from music. Silverman (2010, 2012) described that prior studies on musical mnemonics focused on familiar types of verbal information (i.e., multiplication tables, phone numbers, random numbers and types of text) and unfamiliar and novel types of verbal information in various populations (i.e., young children, children diagnosed with learning impairment or with cognitive impairment, persons with Multiple Sclerosis (MS), dysphasia, and nursing-home residents with memory loss due to Alzheimer’s disease (AD)). However, despite the widespread informal use of music as a mnemonic aid in both general and patient populations, the research on this topic in this patient group is still limited (Simmons-Stern et al., 2010) and there is a clear need for future research to unravel mechanisms through which musical mnemonics might aid episodic memory functioning in AD. Furthermore, research in AD to date has mainly focused on the functioning of long-term episodic musical memory (Moussard et al., 2012, 2014; Simmons-Stern et al., 2010, 2012). To our knowledge, no research was reported on the use of musical mnemonics in working memory paradigms in persons with AD, focusing on the ability to keep information active for a brief period of time in order to manipulate it (Baddeley, 2000), or provide additional structure to allow transition to long-term memory for those with impaired working memory (Rainey & Larsen, 2002).

### Music as a Mnemonic Aid: Possible Underlying Mechanisms

Music is not a unitary concept, but is made up of diverse components such as melody and rhythm. These and other single or combined components have been identified as possible facilitating aspects of music as a mnemonic aid. When music is used as a mnemonic, rhythm was found to increase the ability to chunk information in order to increase the likelihood of encoding and recall (Silverman, 2012). Schön et al. (2008) concluded that pitch may even be effective without addition of rhythm. Others concluded that the melody, which also includes pitch structure, is more effective than only rhythmical information (Ludke et al., 2014; Wallace, 1994). In addition, the complete musical context has also been identified as a facilitating aspect of music as a memory enhancer. Schellenberg and Moore (1985) for example, found that the complete musical context, including pitch (e.g., scale, mode, contour) and rhythm (e.g., beat, meter) contributed to a meaningful musical context, making a passage easier to learn. They also proposed that pitch and rhythm are two aspects of an interactive system, and that removal of one of these parameters might strongly weaken the meaningful context, or the aiding component. McElhinney and Annett (1996) concluded that the integration of text, melody and rhythm, provided by the musical presentation, could have promoted better organization of information and thus might have enhanced recall. The relevance of the complete context is also supported by the notion of a “joint accent structure” in music (Jones, 1987), that is an integrated combination of the pattern of perceptual accents in pitch, rhythm, and other musical characteristics, that can function as cues for memory by inducing enhanced attention to specific time points in the music. Rainey and Larsen (2002) suggest that a basis to predict successful memory enhancement through music can be derived from research findings on the storing process of the music and lyrics of songs (separately, or integrated in a single representation).

In their review on the effects of music on verbal learning and memory, Ferreri and Verga (2016) discussed several potential mechanisms. First, music may function as a temporal scaffold, thereby selectively directing attention, and thus reinforce and facilitate learning and memory. Next, music enhances arousal and mood, which has been shown to benefit aspects of cognitive function. Finally, music may activate the reward system through induction of emotional responses. Ferreri and Verga (2016) were the first to review studies on the specific benefits of music on verbal learning and memory, dividing them into studies using a ‘sung vs. spoken’ encoding paradigm or those using background music. They furthermore proposed a model on effects of music on learning and memory in order to explain how different mechanisms might be involved in the previously described paradigms (i.e., sung vs. spoken or background music).

Ferreri and Verga (2016) hypothesized that recruitment of these different cognitive mechanisms (i.e., temporal scaffolding, arousal-mood, emotions-reward) critically depends on the complexity of the musical stimulus such as tempo, mode, arousal, and length, and the experimental paradigm used (sung vs. spoken or background music). This results in either a direct action of the musical stimulus on the verbal material (i.e., temporal scaffolding mechanisms allow anchoring between the verbal and musical stimulus thus resulting in attention direction and possible improvement of memory performance) or, with more complex musical stimuli (e.g., classical background music) in an indirect action via general-purpose mechanisms (attention, arousal-mood, emotions-reward). Finally, Ferreri and Verga (2016) mentioned that their model does not consider familiarity of the melody, but they argued that it possibly could modulate the proposed combined effects of the musical and verbal stimulus.

Emotion and general arousal have also been suggested as a possible mechanisms for enhanced verbal memory seen in an AD population (Moussard et al., 2012; Ratovohery et al., 2019). Another notion, put forward by Moussard et al. (2012), is that shared syntactic processes for music and language may aid memory for songs in AD through enhanced connections between the melody and the lyrics. Finally, Ratovohery et al. (2019) also discussed the deeper and richer encoding (Craik & Lockhart, 1972), and the role of the spared musical processing in AD in contrast with language processing deficits. Furthermore, they noted that aspects inherent to music such as complexity, tempo and harmonic structure, may also contribute to the assumed effect of music as a mnemonic aid.

## Aim of Our Systematic Review

Here, we examined whether the use of musical mnemonics (i.e., sung presentation of verbal information) leads to enhancement of working and episodic memory performance in both cognitively unimpaired individuals and in patients with AD (in which working or episodic memory impairments typically occur, Kessels et al., 2015; Kirova et al., 2015). We performed a systematic review anticipating that most studies would have small sample sizes, have heterogeneous and varying methodological approaches, and without standardized outcome measures precluding formal quantitative meta-analysis. Also, we explored which aspects of music may be relevant in memory enhancement (e.g., familiarity of the melody) and where possible, also taking into account the effect of musical expertise (i.e., an umbrella term referring to musical background and training of the participants, operationalized in different ways in the included studies, ranging from regular informal music activities to formal music studies or professional musicianship) on degree of benefit of musical mnemonics. We synthesized our findings into a theoretical account of the underlying mechanism building on the model of Ferreri and Verga (2016), to help set up a framework for future empirical studies to clarify how music (i.e., aspects of the musical stimulus, stimulus complexity, paradigm) could contribute to the processes of memory in terms of encoding, maintenance and retrieval, also taking into account personal aspects (e.g., cognitive ability, musical expertise). Finally, we provided recommendations for future research through a list of guidelines of what specific information future researchers should report regarding the musical and verbal stimulus and for clinical use (e.g., for memory rehabilitation in people with cognitive impairments including mild cognitive impairment (MCI) and AD).

## Methods

### Search Strategy

A systematic search of the literature through the following *information sources*, that is, the PubMed and PsycINFO databases simultaneously, was completed on May 9, 2022, using a *search strategy* with combinations of the following search terms (or truncated versions): ‘music’, ‘working memory’, or ‘episodic memory’, in accordance with the PRISMA guidelines (Page et al., 2021a, b; Page et al., 2021a, b)(See supplementary materials for our PRISMA checklist). Because of the limited amount of literature on musical mnemonics, we decided not to narrow the search results in advance by already searching with the search terms MCI and AD. As we did not find studies on musical mnemonics in persons with MCI, we here describe the results in the general population and for those with AD.

### Study Selection

For this review only original research articles published in scientific journals were selected when the following *eligibility criteria* were met, namely: a) using musical mnemonics in an experimental setting, and b) measuring the performance on a memory test (i.e., a test measuring a specific memory process such as encoding, retrieval or recall) as an outcome measure. Musical mnemonics were defined as a musical presentation (i.e., sung (using pitch) digits or words). Furthermore, when musical expertise (umbrella term referring to musical background and training of the participants, ranging from regular informal music activities to formal music studies or professional musicianship, specified in various ways in different papers) was included as a covariate these results were also reviewed. Reviews (or articles) not containing original data, studies not published in English, studies published before 1970, studies concerning music therapy not specifically aimed at remembering verbal material, or using music as a context, studies on evoked musical autobiographical memories, studies on tonal working memory, patient studies that did not focus on MCI or AD, and animal studies were excluded. Regarding the *selection process*, first, these criteria were examined by careful screening of the titles and abstracts by one author (MWD, with assistance from RPCK). Subsequently the full-text papers were screened to assess whether they met our inclusion criteria by one author (MWD, with help from RPCK). For each identified paper in the review, the reference list was also manually extracted to identify additional articles. Finally the reference lists of the additional articles were also manually extracted to identify additional articles by one author (MWD, with assistance from RPCK). The *data collection process* consisted of collection of the data from the included reports by one author (MWD) with critical input from RS and RPCK. No tools on *study risk of bias assessment* were used.

### Data-analysis

For each paper only the paradigms of interest (i.e., comparing the performance on a verbal memory test after musical vs. spoken presentation as *data items*) were considered in accordance with our inclusion criteria (for instance, when papers reported multiple experiments) and the corresponding *effect sizes* were recorded. We collected data on the report (e.g., author, year), participant characteristics (i.e., population, number of participants and age (mean and standard deviation/range), musical expertise) and the research design (item characteristics, i.e., materials for memorization and musical stimulus embedding (paradigm, learning phase and testing phase), and memory domain). If data were missing (for example regarding age), this was noted with ‘not reported’ (N.R.)(See Table 1). For the studies included in which effect sizes were not reported, effect sizes (Cohen’s *d*) were computed based on the available data comparing the intervention (i.e., sung) versus control (e.g., spoken or for example rhythmically spoken) conditions. Furthermore, when other effect sizes or statistics were reported, we converted them into Cohen’s *d*, where possible, using available calculators (Lakens, 2013; Lenhard & Lenhard, 2016; Lin, n.d.; Lipsey & Wilson, 2001; Uanhoro, 2017). We interpreted Cohen’s *d* in line with common guidelines (i.e., 0.2, small; 0.5, medium; 0.8 large) (Cohen, 1992). The study effect sizes are listed in Table 1. If possible, the effect sizes were averaged across sub-experiments, but when different paradigms were used within a study for different sub-experiments, the effect sizes were calculated separately.

**Table 1:** Characteristics of the Studies Examining Effects of Music on Memory Functioning

**Article**	**Participant characteristics**				**Item characteristics**					**Memory domain**	**Summary music effect**	**Effect sizes** **(Cohen’s *d*)**
	**Population**	***N* of participants**	**Age** ***M* (*SD*)** **[Range]**	**Musical** **expertise**	**Materials for** **memorization**	**Musical stimulus embedding**						
						Paradigm	Learning phase		Testing phase			
							Familiarity		Modality			
Baird et al., 2017	AD	5	79.0 (11.1)	Mu	Sentences: day, time, task	Sung vs. spoken	F		Spoken + passive Re	EM	M- AD NMu	-0.84
		6	72.5 (7.7)	NMu								
	OA	15	74.9 (7.3)	Mu							ME + AD	0.99
		7	70.0 (1.6)	NMu								
Calvert & Billingsley, 1998	Exp. 2: Ch	39	4.7	N.R	Exp. 2: Telephone number	Exp. 2: Sung / prose / combination	Exp. 2: UF		Exp. 2: Spoken	WM	Exp. 2: M-	-1.24
Calvert & Tart, 1993	Exp. 2: YA	28	19.6	N.R	Prose	Sung vs. spoken	F		Written	WM & EM	Exp. 2: M = SE STR & LTR	0.17
											Exp. 2: M + RE STR & LTR	1.63
Chazin & Neuschatz, 1990	Ch	26	8	N.R	Mineral names	Sung vs. spoken	F		Written	WM & EM	M + IR	0.60
	YA	20	[18–21]								M = DR	0.00
Deason et al., 2012	OA	12	76.3 (7.7)	NMu ME (y/n)	Lyrics	Sung vs. spoken	UF		Passive Re	EM	M =	0.69
Gfeller, 1983	Ch	30	9–11	N.R	Multiplication tables	Sung vs. spoken	UF		Written	WM	M- SR	-0.48
	Ch learning impairment	30	9–11								M + ER	1.72
Good et al., 2015	Spanish-speaking Ch	38	9–13	Some ME	Novel English song lyrics	Sung (Acc guitar) vs. spoken poem		F (UF repetition)	Sung or spoken	EM	M + IR	1.84
											M + IT	0.77
											M + IP	1.86
											M + DR	1.92
											M = DT	0.96
Jellison, 1976	YA	34	N.R	ME/NME (17/17)	Digit span	Sung vs. spoken		UF	Written	WM	M +	1.22
											ME +	0.87
Jellison & Miller, 1982	YA	46	N.R	ME/NME (23/23)	Digit span Word span and/or pitch sequences	Sung vs. spoken		UF	Sung or spoken	WM	M- DS	I.D
											M = WS	I.D
Kilgour et al., 2000	YA	Exp. 1: 78	19.8 (3.2)	ME/NME (39/39)	Lyrics	Exp. 1: Sung vs. sung with piano prelude or spoken		UF	Spoken	EM	Exp. 1: M + IR & DR	0.75
		Exp. 2: 40	20.4 (3.2)			Exp. 2: Sung vs. spoken equated presentation rate					Exp. 2: M-	-0.81
		Exp. 3: 120	19.3 (2.4)			Exp. 3: Sung vs. spoken slow, medium or fast presentation rate					Exp. 3: M-	-0.32
Lehmann & Seufert, 2018	YA	108	16.2 (1.3) [12–19]	ME NGD	Six rhymed verses and a refrain	Sung (accompanied by monophonic piano) vs. spoken or visual		UF	Spoken + C	EM	M– R (vs. visual)	-0.78
											M = R (vs. spoken)	I.D
											M + C (vs. visual)	0.40
											M = C (vs. spoken)	I.D
Ludke et al., 2014	YA	60	21.7 [18–29]	N.R	PAP English & Hungarian	Sung vs. spoken or rhythmic spoken		UF	Spoken + Passive Re	EM	M + IR & DR	0.49
Ma et al., 2020	YA	42	19.3 [17–22]	NMu	Chinese words	Sung vs. spoken (IDS or ADS)		UF/F	Passive Re	EM	M + WL (vs. ADS) Sung = IDS	0.64
											M + DR (vs. ADS) Sung = IDS	0.47
McElhinney & Annett, 1996	YA	20	21.9	N.R	Lyrics	Sung vs. spoken		UF	Written	EM	M = SE	0.56
											M + RE (trial 2 & 3)	2.15
Moussard et al., 2012	Mild AD	1	68	NMu	Lyrics	Sung vs. spoken		UF/F	Sung or spoken	EM	M- IL UF	I.D
											M + RL UF + F	I.D
Moussard et al., 2014	AD	8	77.8 (5.2)	NMu	Lyrics	Sung vs. spoken		UF/F	Sung or spoken	EM	M = IR	I.D
	OA	7	75.7 (7.4)								M + DR: OA F AD UF + F	I.D
Oostendorp & Montel, 2014	AD	21	N.R	N.R	Word list	Sung vs. spoken		F	Sung or spoken	EM	M + CR & FR	I.D
Palisson et al., 2015	AD	12	82.8 (8.9)	ME NGD (high ME excluded)	Text	Sung melody + IA vs. spoken/SME		F	Spoken	EM	M + IR (vs. spoken)	1.13#
											M + IR (vs. SME)	0.87#
	OA	15	77.1 (7.2)								M + DR (vs. spoken)	0.78#
											M + DR (vs. SME)	0.30#
Prickett & Moore, 1991	AD	10	75 [69–87]	N.R	Life-long F material (songs/psalm) vs. first presented material	Sung vs. spoken/rhymed speech		UF/F	Sung and spoken	EM	M + *	I.D
Purnell-Webb & Speelman, 2008	YA	Exp. 1: 100	17–52	ME NGD	Poetry verses	Sung (F/UF melody/unknown/known Rhy) vs. spoken		UF/F	Written	EM	Exp. 1: M + Rhy	I.D
Racette & Peretz, 2007	YA	Exp. 1: 36	25 [20–37]	ME/NME (18/18)	Lines French folksongs	Sung vs. spoken (melody in background)		UF	Sung (on melody) or spoken (lyrics alone)	EM	Exp. 1: IR & DR M =	0.31
Rainey & Larsen, 2002	Exp. 1: YA	79	19.7	N.R	Names sport players	Sung ± PA vs. spoken		F	Spoken	EM	Exp. 1: M = IL	0.06
											Exp. 1: M + RL	0.47
	Exp. 2: YA	102	19.5		Fictional names	Sung vs. spoken/visual presented					Exp. 2: M = IL (vs. spoken)	0.44
											Exp. 2: M + RL	1.43
Ratovohery et al., 2018	YA	24	22.8 (2.0)	ME NGD	Text	Sung on melody of instrumental music (PV/NV) vs. spoken		F	Spoken	EM	M + PV OA (IR & DR (10 m & 24 h)	0.56
	OA	30	75.5 (6.9)									
Ratovohery et al., 2019	AD	13	77.9 (8.1)	LME	Text everyday life	Sung on melody of instrumental music (PV/NV) vs. spoken		F	Spoken	EM	M + AD (Encoding & IR & DR (10 m &24 h)	1.14
	OA	26	77.1 (8.2)									
Rukholm et al., 2018	YA	66	[17–30 +]	N.R	Lyrics	Sung vs. spoken (poem) & amount of elaboration (LE/HE)		F	Written	EM	M + HE receptive learning	I.D
											M + HE productive learning	I.D
Schön et al., 2008	Exp. 1: YA	26	23	NMu	Nonsense words	Exp. 1: Spoken		UF	Passive Re	EM	M + Exp. 2 vs. Exp. 1	1.45
	Exp. 2: YA	26	23			Exp. 2: Sung (constant syllable-pitch matching)					M + Exp. 3 vs. Exp. 1	0.72
	Exp. 3: YA	26	23.5			Exp. 3: Sung (variable syllable-pitch mapping)						
Silverman, 2007	YA	120	N.R	ME/NME (72/48)	Digit span	Sung vs. spoken (Pitch/Rhy/Pitch & Rhy)		UF	Written	WM	M +	0.71
Silverman, 2010	YA	60	N.R	ME/NME (30/30)	Digit span	Sung (Pitch/Rhy/Pitch & Rhy)		UF/F	Written	WM	M + Rhy > Pitch, Pitch & Rhy	0.67†
Silverman, 2012	YA	60	N.R	ME/NME (30/30)	Digit span	Sung (Melodic complexity/Rhy/NRhy)		UF	Written	WM	M + Rhy > NRhy	1.57†
Silverman & Schwartzberg, 2014	YA	60	22.9 (5.8)	ME (30)	Digit span	Sung (Fe/M voice) & NA/Acc (piano, guitar)		UF	Written	WM	M + M > Fe voice,	0.58†
			21.1 (3.0)	NME (30)							M + piano, N Acc > guitar	1.01†
Silverman & Schwartzberg, 2019	YA	60	N.R	ME/NME (30/30)	Digit span	Sung (V + Au/Au) vs Spoken (V + Au/Au) vs Melody (V + Au/Au)		UF	Written	WM	V + Au: M + Sung	0.55
											V + Au: M + Melody	0.47
Simmons-Stern et al., 2010	AD	13	77.3 (7.6)	NMu + ME (y/n)	Lyrics excerpts	Sung vs. spoken		UF	Passive Re	EM	M + AD	1.07
	OA	14	73.7 (5.5)								M = OA	0.35
Simmons-Stern et al., 2012	AD	12	81.2 (4.0)	ME NGD	Novel lyrics	Sung vs. spoken		UF	Passive Re	EM	M + GC	0.80
	OA	12	78.6 (8.7)								M = SC	0.00
Tamminen et al., 2017	Students/ staff	Exp. 1: 39	21	NMu	Novel words	Exp. 1: Spoken		UF/F	Spoken & passive measures	EM	M = R Exp. 2 & 3 vs. Exp. 1	I.D
		Exp. 2: 39	21			Exp. 2: Sung UF					M = Re Exp. 2 & 3 vs. Exp. 1	I.D
		Exp. 3: 39	20			Exp. 3: Sung F					M + IML F Exp. 3 vs. Exp. 1	0.70
Wallace, 1994	Exp. 1: YA	64	N.R	Some ME	Text	Exp. 1: 3 vs Sung (OM) vs. spoken		UF	Written	EM	Exp. 1: M +	1.11
	Exp. 2: YA	21	N.R			Exp. 2: 3 vs Sung (OM) vs. rhythmic spoken					Exp. 2: M +	0.54
	Exp. 3: YA	39	N.R			Exp. 3: 1 vs Sung vs. spoken					Exp. 3: M-	-0.60
	Exp. 4: YA	48	N.R			Exp. 4: 3 vs Sung (OM) vs. 3 vs Sung (DM) vs. spoken					Exp. 4: M +	0.83
Wolfe & Hom, 1993	Ch	10	5	N.R	Telephone numbers	Sung vs. spoken with(out) contingent music		UF/F	Spoken	WM & EM	M + L	1.00
											M = IR	I.D
											M = Ret	I.D
Yalch, 1991	Exp. 2: YA	124	N.R	N.R	Exp. 2: Soundtrack television commercials	Exp. 2: Jingle vs. no jingle Number of exposures		N.R	AR & Passive Re	EM	Exp. 2: M + AR & Re	1.21

Articles listed in alphabetical order. If the effect concerned both groups this is not specified, if an effect concerned one of the groups this is separately mentioned in the ‘summary of music effect’ column. Positive effect sizes indicate an advantage of the condition of interest versus spoken (unless otherwise specified†). Abbreviations in alphabetical order: *Acc* accompaniment, *AD* Alzheimer’s dementia, *ADS* adult directed speech, *AR* aided recall, *Au* auditory, *C* comprehension, *Ch* children, *CR* cued recall, *DM* different melodies, *DR* delayed recall, *DS* digit span, *DT* delayed translation, *EM* episodic memory, *ER* extended rehearsal, *Exp*. experiment, *F* familiar, *Fe* female, *FR* free recall, *GC* general content, *HE* high elaboration, *IA* instrumental accompaniment, *I.D.* insufficient data reported to compute the (parametric) effect size, *IDS* infant directed speech, *IL* initial learning, *IML* integration mental lexicon, *IP* immediate production, *IR* immediate recall, *IT* initial translation, *L* learning, *LE* low elaboration, *LME* low musical expertise, *M* male, *ME* musical expertise, *ME* + musical experts perform better on a sung presentation than persons with less musical expertise, *Mu* musicians, *M* = no difference between a sung or spoken presentation, *M-* sung < spoken, *M* + sung > spoken, *N* number of participants, *NA* no accompaniment, *NGD* no group differences, *NME* no musical expertise, *NMu* non-musicians, *NV* negative valence, *N.R.* not reported, *NRhy* no rhythm, *OA* (cognitively unimpaired) older adults, *OM* one melody, *PA* piano accompaniment, *PAP* paired associate phrases, *PV* positive valence, *R* recall, *Re* recognition, *RE* repeated exposure, *Ret* retention, *RL* relearning, *Rhy* rhythm, *SC* specific content, *SE* single exposure, *SME* silent movie excerpts, *SR* single rehearsal, *UF* unfamiliar, *V* visual, *vs* verses, *vs.* versus, *WL* word learning, *WM* working memory, *WS* word span, *YA* (cognitively unimpaired) young adults, # Hedges’*g*, * for this study the content of the verbal information differed across conditions

## Results

### Study Characteristics

The search resulted in a total of 1,126 articles published between 1971 and 2022. A total of 1,091 articles were excluded after reviewing the titles and abstracts for eligibility. Full-text articles were retrieved for 35 studies, 14 of which were eligible for inclusion. Forty-seven additional studies were assessed after searching the reference lists, of which 17 additional articles were eligible for inclusion. Finally, fourteen additional studies were assessed after searching the references lists of the additional studies included, of which six articles were eligible for inclusion, which resulted in a total of 37 included papers.

**Fig. 1:**
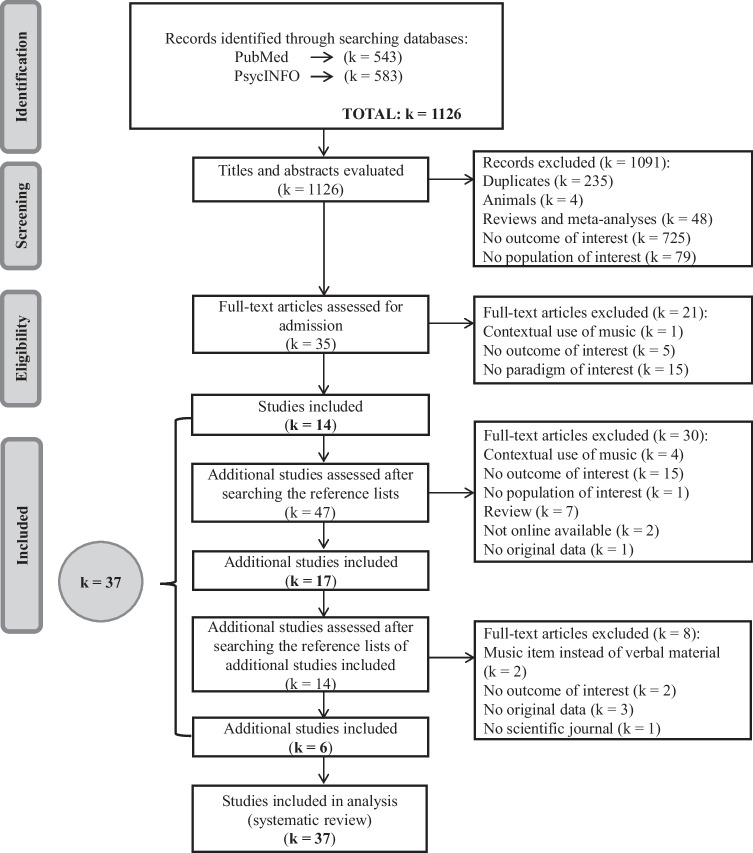
Flowchart of Literature Search. This flowchart represents the search completed on May 9, 2022. k = number of studies

Figure 1 depicts the flowchart of this search. Regarding the *results of the individual studies*, Table 1 shows the characteristics of the included papers (see also Supplementary Table 1 which provides more detailed information about the differences in learning phase, as well as a more elaborate description of the included studies). In the reference list studies with an asterisk reflect that they were included in this systematic review.

### Participant Characteristics

Twenty studies regarding the use of music as a mnemonic aid were performed in young adults (see Table 1 for the demographic variables, including age) (Calvert & Tart, 1993; Jellison, 1976; Jellison & Miller, 1982; Kilgour et al., 2000; Lehmann & Seufert, 2018; Ludke et al., 2014; Ma et al., 2020; McElhinney & Annett, 1996; Purnell-Webb & Speelman, 2008; Racette & Peretz, 2007; Rainey & Larsen, 2002; Rukholm et al., 2018; Schön et al., 2008; Silverman, 2007, 2010, 2012; Silverman & Schwartzberg, 2014, 2019; Wallace, 1994; Yalch, 1991), one study used students and university staff (Tamminen et al., 2017), and five studies were conducted in children (aged 4–13) (Calvert & Billingsley, 1998; Chazin & Neuschatz, 1990; Gfeller, 1983; Good et al., 2015; Wolfe & Hom, 1993). One of these compared learning-impaired elementary school children (with reading, math or written language difficulties) and typically developing children (Gfeller, 1983) and one included elementary school children and young adults (Chazin & Neuschatz, 1990).

In total, eight studies included cognitively unimpaired older adults (all aged above 65). Two of these studies focused only on cognitively unimpaired older adults (Deason et al., 2012; Ratovohery et al., 2018), and six studies on AD had a control group consisting of cognitively unimpaired older adults (Baird et al., 2017; Moussard et al., 2014; Palisson et al., 2015; Ratovohery et al., 2019; Simmons-Stern et al., 2010, 2012).

Nine studies regarding the use of music as a mnemonic aid have been conducted in persons diagnosed with AD (Baird et al., 2017; Moussard et al., 2012, 2014; Oostendorp & Montel, 2014; Palisson et al., 2015; Prickett & Moore, 1991; Ratovohery et al., 2019; Simmons-Stern et al., 2010, 2012). No studies on musical mnemonics in persons with MCI were found.

### Materials for Memorization

In all of the studies, participants were asked to remember verbal information. When working memory was assessed, researchers used digit span paradigms (Jellison, 1976; Jellison & Miller, 1982; Silverman, 2007, 2010, 2012; Silverman & Schwartzberg, 2014, 2019), word span tasks (Jellison & Miller, 1982), multiplication tables (Gfeller, 1983), mineral names (Chazin & Neuschatz, 1990), and a telephone number (Calvert & Billingsley, 1998; Wolfe & Hom, 1993).

When episodic memory was assessed, studies used novel types of information such as fictional names (Rainey & Larsen, 2002), nonsense words (Schön et al., 2008; Tamminen et al., 2017), word lists (Oostendorp & Montel, 2014), ballad verses (Wallace, 1994), text (Palisson et al., 2015), poetry verses (Purnell-Webb & Speelman, 2008), excerpts of unfamiliar folk songs (Racette & Peretz, 2007), lyrics (Deason et al., 2012; Kilgour et al., 2000; McElhinney & Annett, 1996; Moussard et al., 2012, 2014), lyrics excerpts (Simmons-Stern et al., 2010), novel lyrics about activities of daily living (Simmons-Stern et al., 2012), sentences relevant to daily life of older adults (Baird et al., 2017), text about everyday themes (Ratovohery et al., 2018, 2019), a foreign language (Good et al., 2015; Ludke et al., 2014; Ma et al., 2020; Rukholm et al., 2018), lifelong familiar material in comparison to firstly presented material (Prickett & Moore, 1991), advertisement slogans (Yalch, 1991), and prose (Calvert & Tart, 1993).

### Musical Stimulus Embedding

Almost all studies compared a sung versus spoken (or combined) presentation of stimuli, but considerable differences exist between the different paradigms. In the majority of research, the participants took part on an individual basis (except in the studies of Calvert & Tart, 1993; Calvert & Billingsley, 1998; Good et al., 2015; Lehmann & Seufert, 2018; McElhinney & Annett, 1996; Rukholm et al., 2018, and Yalch, 1991, who used (small) groups). Most research (except the studies by Good et al., 2015; Oostendorp & Montel, 2014; Prickett & Moore, 1991, and Wolfe & Hom, 1993) used prerecorded sound files of male or female singers, with sufficient experience in singing or professional singers. In most of the paradigms, at encoding, participants only listened to a musical presentation of information, but in some studies they actively participated by singing the to-be-learned information themselves (e.g., Chazin & Neuschatz, 1990; Good et al., 2015; Oostendorp & Montel, 2014; Prickett & Moore, 1991). Some researchers compared a rhythmical, melodic or combined presentation to a spoken presentation (e.g., Ludke et al., 2014; Purnell-Webb & Speelman, 2008; Silverman, 2007, 2010; Wallace, 1994). Six studies considered or matched the presentation rate of sung or spoken material (Baird et al., 2017; Good et al., 2015; Kilgour et al., 2000; Lehmann & Seufert, 2018; Ludke et al., 2014; Ma et al., 2020). In several studies the verbal material was presented bimodally at encoding (i.e., visually and auditory), for example in the study of Rainey and Larsen (2002). Others used also visually presented text accompanied by a sung or spoken presentation (Calvert & Billingsley, 1998; Calvert & Tart, 1993; Deason et al., 2012; Good et al., 2015; Ma et al., 2020; Ratovohery et al., 2018, 2019; Rukholm et al., 2018; Simmons-Stern et al., 2010, 2012). Silverman and Schwartzberg (2019) compared a visual and auditory versus only auditory presentation. Lehmann and Seufert (2018) compared learning of a text in three modalities (i.e., visual, sung or spoken).

Other aspects of the musical stimulus embedding that have been considered are melodic complexity, variable vs. constant syllable mapping, vocalization type, voice timbre (male/female) and type of instrumental accompaniment, musical valence, i.e., the emotional value of music (positive or negative affect), and the familiarity of the melodies (Ludke et al., 2014; Ma et al., 2020; Ratovohery et al., 2018, 2019; Schön et al., 2008; Silverman, 2012; Silverman & Schwartzberg, 2014).

Some authors systematically investigated the effect of familiarity of the melody (Ma et al., 2020; Moussard et al., 2012, 2014; Prickett & Moore, 1991; Purnell-Webb & Speelman, 2008; Silverman, 2010; Tamminen et al., 2017; Wolfe & Hom, 1993). Other researchers only used unfamiliar melodies (Deason et al., 2012; Gfeller, 1983; Jellison, 1976; Jellison & Miller, 1982; Kilgour et al., 2000; Lehmann & Seufert, 2018; Ludke et al., 2014; McElhinney & Annett, 1996; Racette & Peretz, 2007; Schön et al., 2008; Silverman, 2007; Silverman & Schwartzberg, 2014; Simmons-Stern et al., 2010, 2012; Wallace, 1994). Finally, some studies only used familiar music (Baird et al., 2017; Calvert & Tart, 1993; Chazin & Neuschatz, 1990; Good et al., 2015; Palisson et al., 2015; Rainey & Larsen, 2002; Ratovohery et al., 2018, 2019; Rukholm et al., 2018) or used a previously learned melody (Oostendorp & Montel, 2014).

In the vast majority of studies participants were tested through spoken or written recall. Jellison and Miller (1982) and Good et al. (2015) gave their participants the choice if they wanted to sing or speak at recall. Some researchers instructed their participants to recall the material preferably in the same modality (i.e., sung or spoken) as they learned the material (Moussard et al., 2012, 2014) following the encoding-specificity principle (i.e., how information can be retrieved depends on how it was stored) (Tulving & Thomson, 1973). Some authors investigated different combinations of modalities of learning (sung versus spoken) and recall (sung versus spoken) (Ludke et al., 2014; Racette & Peretz, 2007). Finally, Lehmann and Seufert (2018) also studied the effect of listening to the previously learned melody while recalling the to-be-learned text.

### Memory Domain

Nine studies focused on working memory (Calvert & Billingsley, 1998; Gfeller, 1983; Jellison, 1976; Jellison & Miller, 1982; Silverman, 2007, 2010, 2012; Silverman & Schwartzberg, 2014, 2019). Of these, Calvert and Billingsley (1998) focused on working memory in pre-school children and Gfeller (1983) focused on elementary school children, while all others focused on young adults. No studies on the effects of a musical presentation on working memory conducted in cognitively unimpaired older adults or persons with MCI or AD were found.

Three studies focused on working memory as well as on episodic memory (Calvert & Tart, 1993; Chazin & Neuschatz, 1990; Wolfe & Hom, 1993). Sixteen of the 25 studies on episodic memory focused on cognitively unimpaired participants (Deason et al., 2012; Lehmann & Seufert, 2018; Good et al., 2015; Kilgour et al., 2000; Ludke et al., 2014; Ma et al., 2020; McElhinney & Annett, 1996; Purnell-Webb & Speelman, 2008; Racette & Peretz, 2007; Rainey & Larsen, 2002; Ratovohery et al., 2018; Rukholm et al., 2018; Schön et al., 2008; Tamminen et al., 2017; Wallace, 1994; Yalch, 1991), of which two focused specifically on episodic memory in older adults without cognitive impairment (Deason et al., 2012; Ratovohery et al., 2018). The other nine included persons diagnosed with AD (Baird et al., 2017; Moussard et al., 2012, 2014; Oostendorp & Montel, 2014; Palisson et al., 2015; Prickett & Moore, 1991; Ratovohery et al., 2019; Simmons-Stern et al., 2010, 2012). Most studies used immediate and recall measures, a few researchers used recognition measures (Deason et al., 2012; Ma et al., 2020; Schön et al., 2008; Simmons-Stern et al., 2010, 2012), and some both (Baird et al., 2017; Tamminen et al., 2017; Yalch, 1991). Some authors used both free and cued recall measures (Oostendorp & Montel, 2014). Finally, some authors also included comprehension measures (Lehmann & Seufert, 2018).

### Synthesis of Findings

Overall, 28 out of 37 studies found that a musical (i.e., sung) presentation had a beneficial effect on some aspect of memory performance (seven out of nine studies concerning working memory: Gfeller, 1983; Jellison, 1976; Silverman, 2007, 2010, 2012; Silverman & Schwartzberg, 2014, 2019, and twenty-one out of 28 studies concerning episodic (or working and episodic) memory: Calvert & Tart, 1993; Chazin & Neuschatz, 1990; Good et al., 2015; Ludke et al., 2014; Ma et al., 2020; McElhinney & Annett, 1996; Moussard et al., 2012, 2014; Oostendorp & Montel, 2014; Palisson et al., 2015; Prickett & Moore, 1991; Purnell-Webb & Speelman, 2008; Rainey & Larsen, 2002, Ratovohery et al., 2018, 2019; Rukholm et al., 2018; Schön et al., 2008; Simmons-Stern et al., 2010, 2012; Wallace, 1994; Yalch, 1991).

However, some of these authors did not find an effect on other aspects of the included measures (Calvert & Tart, 1993; Chazin & Neuschatz, 1990; Good et al., 2015; McElhinney & Annett, 1996; Moussard et al., 2014; Rainey & Larsen, 2002; Simmons-Stern et al., 2012; Yalch, 1991), participant groups (Simmons-Stern et al., 2010), or even a partly detrimental effect (Gfeller, 1983; Moussard et al., 2012; Wallace, 1994).

Nine studies did not find overall effects of a musical presentation on memory performance (working memory: Calvert & Billingsley, 1998; Jellison & Miller, 1982, episodic memory: Baird et al., 2017; Deason et al., 2012; Lehmann & Seufert, 2018; Kilgour et al., 2000; Racette & Peretz, 2007; Tamminen et al., 2017; Wolfe & Hom, 1993). As before, some of these authors did however find positive effects in a part of their experiments (Calvert & Billingsley, 1998; Kilgour et al., 2000) or in other cognitive measures (Lehmann & Seufert, 2018; Tamminen et al., 2017; Wolfe & Hom, 1993) and in comparison to another control condition than spoken presentation (Lehmann & Seufert, 2018). Finally here too, some authors found a detrimental effect in a part of their experiments (Calvert & Billingsley, 1998; Jellison & Miller, 1982; Kilgour et al., 2000) or in a part of the participant groups (Baird et al., 2017). Thus, in general beneficial effects of musical mnemonics on some aspect of memory performance were reported in children, young and older adults with and without memory impairment, however a minority of studies found no overall effect. In the following sections, these results are discussed in more detail, starting with the results summarized by participant population (children, cognitively unimpaired young adults, older adults, AD, musical expertise of the participants) followed by discussion of the results summarized by aspects of the musical stimulus embedding (i.e., melody, rhythm, participation at encoding, familiarity, and other variables) (see also Table 1 and Supplementary Table 1 in the supplementary materials for more details about specific effects of the included studies).

#### Children and Cognitively Unimpaired Young Adults

Twenty-eight studies focused on cognitively unimpaired participants. Five studies conducted in children showed mixed results concerning different stages of memory; Gfeller (1983) found that musical rehearsal together with modeling and cueing significantly aided retention of sung information in both typically developing and learning-impaired students. Chazin and Neuschatz (1990) found only a benefit at immediate recall of musically presented mineral names; Wolfe and Hom (1993) found that a sung presentation of a telephone number at initial learning resulted in fewer learning trials in young children, however, Calvert and Billingsley (1998) found in one of two experiments that young children remembered their telephone numbers best when presented in prose relative to a song. Good et al. (2015) found that Spanish-speaking children (aged 9–13) who learned a novel sung English passage for two weeks, outperformed children who learned the passage presented as an oral poem (i.e., on verbatim recall, pronunciation, translation). Furthermore, the recall advantage of the sung presentation still existed at very long-term recall (six months). Most of the studies performed in young adults found a significant effect of a musical presentation of information to enhance aspects of memory performance (Calvert & Tart, 1993; Jellison, 1976; Ludke et al., 2014; Ma et al., 2020; McElhinney & Annett, 1996; Purnell-Webb & Speelman, 2008; Rukholm et al., 2018; Schön et al, 2008; Silverman, 2007, 2010, 2012; Silverman & Schwartzberg, 2014, 2019; Wallace, 1994; Yalch, 1991). Rainey and Larsen (2002) found no significant effect at initial learning; however, they did find that relearning the word list a week later required fewer trials in the sung version.

Others found no significant effect (Jellison & Miller, 1982; Racette & Peretz, 2007; Tamminen et al., 2017), or a partly detrimental effect (Jellison & Miller, 1982, only for digit span). Kilgour et al. (2000) initially found an effect of a sung presentation which reversed after controlling for presentation rate. Tamminen et al. (2017) on the other hand, failed to find effects on memory but did find effects of a sung presentation on learning. In line with this, Lehmann and Seufert (2018) also did not find any effects of sung versus spoken presentation on text recall, but only demonstrated an effect on comprehension (sung vs. visual presentation).

To conclude, only a few researchers have investigated the use of musical mnemonics in children, showing mixed results, while the majority of research in cognitively unimpaired participants that focused on young adults generally showed beneficial results.

#### Cognitively Unimpaired Older Adults

Of the 28 studies using cognitively unimpaired participants, two focused specifically on cognitively unimpaired older adults (Deason et al., 2012; Ratovohery et al., 2018). Deason et al. (2012) did not find a significant benefit in recall of sung lyrics (even) after a one-week delay (to avoid a ceiling effect), in contrast with persons with AD (from the study of Simmons-Stern et al., 2010), whose memory performance was enhanced by musical encoding. Deason et al. (2012) concluded that maybe there is a fundamental difference in musical encoding between older adults without cognitive impairments and those with AD. Ratovohery et al. (2018) on the other hand, found a significant better recall of sung lyrics in cognitively unimpaired older adults. This result however was only found when the music was positively valenced in terms of emotional content, regardless of the retention delay.

Interestingly, six of the studies on AD included a control group consisting of matched cognitively unimpaired older adults. Simmons-Stern et al. (2010) did not find a benefit of a sung presentation of lyrics, and Baird et al. (2017) also failed to find a significant effect of a sung versus spoken presentation in cognitively unimpaired musicians and non-musicians, but here before the last learning trials all cognitively unimpaired older adults reached errorless performance, indicating a ceiling effect. Additionally, there were no significant differences found between musicians or non-musicians for any of the experimental task variables.

The other four studies demonstrated a significant effect of a sung presentation in cognitively unimpaired older adults. Simmons-Stern et al. (2012) found a benefit of recall of sung lyrics concerning general content. Moussard et al. (2014) only showed a significantly improved delayed (but not immediate) recall, while Palisson et al. (2015) reported a significantly improved immediate and delayed recall, and Ratovohery et al. (2019) found a better recall only for positively valenced music.

In conclusion, research on the effects of musical mnemonics in cognitively unimpaired older adults is scarce. Only recently have some researchers focused on effects of musical mnemonics in older adults, mostly using them as a control group for persons with AD, again showing mixed results.

#### Alzheimer’s Disease

Nine studies focused on the effects of music as a mnemonic device on episodic memory in AD (Baird et al., 2017; Moussard et al., 2012, 2014; Oostendorp & Montel, 2014; Palisson et al., 2015; Prickett & Moore, 1991; Ratovohery et al., 2019; Simmons-Stern et al., 2010, 2012). All studies except one (Baird et al., 2017) reported a beneficial effect of a sung versus spoken presentation on episodic memory functioning in AD.

The first study on musical mnemonics in AD was carried out by Prickett and Moore (1991), who showed that persons with AD recalled long-familiar songs most accurately (compared to new songs, rhymed speech and spoken words). In line with this, Simmons-Stern et al. (2010) found a significant better recognition of sung lyrics in persons with AD, and in follow-up research (Simmons-Stern et al., 2012) improved memory was reported for only general (rather than specific) content in a sung compared to a spoken presentation of novel song lyrics related to instrumental activities of daily living. This study was followed-up by a case study by Moussard et al. (2012) in a person with AD, showing that singing new lyrics significantly improved the free delayed (10 min) and long-term delayed (four weeks) recall of words, albeit after repeated learning trials. Moussard et al. (2014) confirmed their previous findings in a follow-up patient-control study; sung presentation of lyrics only significantly improved delayed (not immediate) recall.

In contrast to Moussard et al., (2012, 2014), Palisson et al. (2015) found that a sung presentation (familiar melody) of texts compared to a non-musical association or spoken presentation not only led to significantly increased delayed, but also immediate recall, relative to a spoken presentation. Finally, Oostendorp and Montel (2014) reported that free and cued recall of word lists significantly improved after sung presentation in persons with moderate to severe AD.

Although research aimed at musical mnemonics in AD showed positive results in general, the research paradigms that have been used vary greatly with respect to musical stimulus embedding, verbal stimulus, test type (recall versus recognition) or delay (immediate versus delayed), as did the participant characteristics. This may explain the heterogeneity of the findings.

#### Musical Expertise

Although musical background and training were operationalized in different ways, we consider them together under the umbrella term expertise. Ten studies included musical expertise as a covariate (Baird et al., 2017; Jellison, 1976; Jellison & Miller, 1982; Kilgour et al., 2000; Racette & Peretz, 2007; Silverman, 2007, 2010, 2012; Silverman & Schwartzberg, 2014, 2019). Performance differences related to musical expertise were found in nine studies, focusing either on generally higher memory performance in musically trained or expert participants (Jellison & Miller, 1982; Kilgour et al., 2000; Silverman, 2007, 2010, 2012; Silverman & Schwartzberg, 2014, 2019), or interactions indicating a larger benefit of musical presentation on memory performance in participants with more musical expertise (Baird et al., 2017; Jellison, 1976).

For student participants, Jellison (1976) found that while song facilitated digit recall in both musically trained and untrained participants, sung presentation led to a consistently better performance for the musically trained group. Other studies on the other hand, did not find a difference between levels of musical expertise of students in terms of the benefits of sung versus spoken presentation (e.g., Jellison & Miller, 1982; Kilgour et al., 2000; Racette & Peretz, 2007). However with regard to verbal recall, Jellison and Miller (1982) found that musically trained participants recalled more words and digits than untrained participants, and Kilgour et al. (2000) also reported that the musically trained participants outperformed those without training. Silverman (2007, 2010, 2012) and Silverman and Schwartzberg (2014, 2019) also repeatedly reported in young adults that musicians tended to outperform non-musicians in overall on working memory tasks.

In previous AD research, some researchers either did not systematically compare participants with different levels of musical expertise (Oostendorp & Montel, 2014; Prickett & Moore, 1991; Simmons-Stern et al., 2010, 2012) or explicitly focused on non-musicians only (Moussard et al., 2012, 2014). However, Baird et al. (2017) specifically directed their research to possible differences in the benefits of a musical mnemonic between musicians and non-musicians (persons with AD and cognitively unimpaired older adults). Baird et al. (2017) reported that AD musicians did not show a difference in memory performance between a sung and spoken presentation (in contrast to AD non-musicians who actually experienced a negative effect). However, compared to non-musicians with AD, musicians with AD performed better in the sung modality. In contrast, Ratovohery et al. (2019) focused specifically on persons with AD and a low musical expertise, and found improvement of text recall of daily-life themes with a sung (regardless of musical valence) presentation. They showed that even after a 24-h delay and the presence of severe memory impairments in persons with AD with low musical expertise, the musical mnemonic was effective.

To summarize, most studies in cognitively unimpaired participants found no evidence for musical expertise modulating the effect of a sung presentation of information, except one (Jellison, 1976). In AD some authors did not systematically compare musical expertise, others included only musically untrained participants, however, one study that included musical expertise as a covariate showed better learning of sung information in AD musicians (Baird et al., 2017).

#### Musical Stimulus Embedding

As mentioned above, almost all included studies compared a sung versus spoken presentation of stimuli (except the studies of Silverman, 2010, 2012, and Silverman & Schwartzberg, 2014).

##### Melody

Some investigators report that melody contributes to the beneficial results: Wallace (1994) found better verbatim immediate and delayed word recall in a sung condition compared to other presentations (among which a rhythmically spoken presentation) thus supporting melody as a memory enhancer for text if the same, simple melody was repeatedly heard. Ludke et al. (2014) also reported benefits of singing (using an unfamiliar melody) immediately and after 20 min delay compared to (rhythmically) speaking on verbatim recall of short-term paired-associated phrase learning in a foreign language (Hungarian) and native language (not explained by presentation rate as this possible confounder was carefully controlled). Similar effects were described by Rukholm et al. (2018) in adults and in children (Good et al., 2015). Schön et al. (2008) found that by constant mapping of melodic information (pitch) to the syllables of to-be-learned new (nonsense) words arousal and boundary enhancement was reached, presumably contributing to speech segmentation in learning a foreign language and concluded that especially in the first learning phase (i.e., where it is needed to segment new words), one may largely benefit from the structural and motivational benefits of melodic information in song.

##### Rhythm

Others have found that specifically rhythm yielded significant positive results compared to a spoken presentation. Purnell-Webb and Speelman (2008) reported that rhythm, as compared to an unfamiliar melody and spoken condition, facilitates verbatim recall of verbal information. Silverman (2007, 2010, 2012) also described a significant effect of rhythmic presentation on working memory functioning as measured by experimental digit span task performance.

##### Participation at Encoding

All studies that included active rehearsal conditions (participants had to sing the to-be-learned information) (Chazin & Neuschatz, 1990; Gfeller, 1983; Good et al., 2015; Ludke et al., 2014; Moussard et al., 2012, 2014; Oostendorp & Montel, 2014; Palisson et al., 2015; Prickett & Moore, 1991; Ratovohery et al., 2018, 2019) showed positive results on some aspect of memory performance (except the study of Racette & Peretz, 2007). In contrast, of the studies where encoding consisted of listening to a sung presentation, 17 out of 25 showed an effect of a sung presentation on some aspect of memory performance (Calvert & Tart, 1993; Jellison, 1976; Ma et al., 2020; McElhinney & Annett, 1996; Purnell-Webb & Speelman, 2008; Rainey & Larsen, 2002; Rukholm et al., 2018; Schön et al., 2008; Silverman, 2007, 2010, 2012; Silverman & Schwartzberg, 2014, 2019; Simmons-Stern et al., 2010, 2012; Wallace, 1994; Yalch, 1991).

##### Familiarity

Previous research considering the familiarity of the melody can be divided into research that systematically investigated the effect of familiarity and research that only used either unfamiliar or familiar melodies. Familiarity contributed positively to this beneficial effect of sung presentation in cognitively unimpaired adults, requiring more extensive investigation in AD. In sum, four of the eight studies that systematically assessed the effect of the familiarity of the melody or rhythm, found a positive effect (Prickett & Moore, 1991; Purnell-Webb & Speelman, 2008; Tamminen et al., 2017; Wolfe & Hom, 1993), four failed to find an effect of familiarity (Ma et al., 2020; Moussard et al., 2012, 2014; Silverman, 2010). In the following we will discuss the findings in the cognitively unimpaired participants first, followed by findings in AD.

In cognitively unimpaired participants, research that systematically evaluated the effects of familiarity of the melody found that a familiar melody or rhythm (i.e., presenting in a temporal pattern including strong and weak beats that complements the natural meter of spoken text, derived from a well-known melody; Purnell-Webb & Speelman, 2008) facilitated learning (Tamminen et al., 2017) or recall (Purnell-Webb & Speelman, 2008; Wolfe & Hom, 1993). However, Silverman (2010), did not find any difference in reduction of the working memory overload when a familiar melody was used, as compared to an unfamiliar melody. Ma et al. (2020) found also no difference in immediate and long-term memory performance between a familiar and unfamiliar melody. Several studies in cognitively unimpaired participants used unfamiliar melodies; nine of these sixteen studies found a positive (or partly positive) result (Gfeller, 1983; Jellison, 1976; Ludke et al., 2014; McElhinney & Annett, 1996; Schön et al., 2008; Silverman, 2007; Silverman & Schwartzberg, 2014, 2019; Wallace, 1994). Seven studies used a familiar melody, of which five studies found a positive (or partly positive) result (Calvert & Tart, 1993; Chazin & Neuschatz, 1990; Good et al., 2015; Ratovohery et al., 2018; Rukholm et al., 2018).

In research on AD, only one of the three studies that systematically compared the familiarity of the melody (Moussard et al., 2012, 2014; Prickett & Moore, 1991) reported that a familiar melody facilitated the recall (Prickett & Moore, 1991). Moussard et al. (2012) found a detrimental effect of an unfamiliar melody at initial learning. Two studies used an unfamiliar melody and found some positive results (Simmons-Stern et al., 2010, 2012), the four remaining studies used a familiar or familiarized (Oostendorp & Montel, 2014) melody, of which three studies found a positive result (Oostendorp & Montel, 2014; Palisson et al., 2015; Ratovohery et al., 2019). Baird et al. (2017) observed no overall effect of a sung presentation using a familiar melody (although AD musicians did benefit compared to AD non-musicians).

To summarize, studies that systematically compared familiarity in cognitively unimpaired participants showed an advantage of a familiar melody (or rhythm). However, many studies used only a familiar or an unfamiliar melody, showing mixed results. In AD some researchers systematically compared familiarity of the melody, others applied either familiar or unfamiliar melodies only, showing mixed results.

##### Other Variables

Other aspects that have been investigated are the singer’s sex, the kind of accompaniment, live or recorded presentation, sensory modality (purely audio or combining or compared with visual embedding), serial position, degree of elaboration of the verbal information, speech register with some melodic features (infant-directed speech), presentation speed, melodic complexity as well as the emotional valence of the music. Silverman and Schwartzberg (2014) compared recorded melodies using female and male voices and three kinds of accompaniment (guitar, piano and no accompaniment) and found that the use of a male voice and piano (or no) accompaniment enhanced recall. Silverman and Schwartzberg (2019) revealed that additional visual input overloaded working memory, thereby worsening the recall. Finally, their overall results indicated that information in primacy and recency positions was best recalled. As mentioned, Ratovohery et al. (2018) investigated the impact of the emotional valence of music in cognitively unimpaired older adults and found that musical encoding enhanced their recall only when positively valenced music was used.

Overall, researchers have come to different conclusions about the contributing factors of music as a mnemonic aid (e.g., rhythm, melody, position of the information, degree of elaboration, speech register with some melodic features, male or female voice, musical accompaniment, live or recorded presentation, sensory modality, emotional valence, active or passive rehearsal), leaving no clear answer other than that it seems that each of these aspects can potentially have an effect, and it is likely that their combination, leading to specific accent structures in the musical stimulus that can direct attention, are important.

## Discussion

This systematic review provides an analysis of the effect of musical mnemonics on memory functioning in children, cognitively unimpaired young and older adults, and persons with AD. Additionally, we aimed to clarify which aspects of music can facilitate memory (e.g., melody, rhythm, familiarity), and consider the possible influence of musical expertise on the degree of benefit of music as a mnemonic aid.

In most studies, a beneficial effect of musical presentation was reported although some studies observed no beneficial effect. The findings in younger participants included a few studies in children showing mixed results, but the majority of research that focused on young adults generally showed beneficial results. Studies focusing on cognitively unimpaired older adults were limited; this group serving primarily as a control for persons with AD. Despite a sparsity of studies, predominantly positive results of a musical presentation on episodic memory functioning have been reported in AD. Researchers used varying paradigms (musical stimulus embedding, verbal material, testing method (e.g., immediate or delayed (cued or free) recall or recognition), and participant characteristics, see Table 1) possibly explaining the heterogeneity of the findings. However, our findings support the notion that in AD, the use of a sung presentation improves episodic memory performance, with only one study reporting no beneficial effect in AD musicians and a detrimental effect in AD non-musicians. Possibly in line with the great variety in research paradigms of the studies included in this systematic review, the effect sizes ranged from medium to large. However, several studies failed to find effects of musical mnemonics, with small effect sizes.

Regarding the relevance of specific musical aspects, it is important to mention that very few studies systematically assessed musical components’ potential to facilitate memory. In previous studies, various musical aspects forming the musical stimulus embedding have been considered. Taken together, researchers have come to different conclusions about the contributing factors of musical mnemonics (e.g., rhythm, melody, primacy or recency positions, visual, auditory or combined presentation, male or female voice, musical accompaniment, emotional valence, active or passive rehearsal, individual or group participation), leaving no clear answer other than that it seems that each of these aspects can potentially have an effect. It is likely that combined accent structures (resulting from a combination of the emphasis in the verbal material and the accents in the music) are important. With regard to visual, auditory or combined presentation, Silverman and Schwartzberg (2019) found that addition of visual input to auditory presentation hampered digit recall performance through possible overload of working memory. With regards to the contribution of the degree of familiarity of the melody, most research in cognitively unimpaired participants did not systematically compare familiar and unfamiliar melodies. A small majority of the studies that systematically compared familiarity reported an advantage of a familiar melody (or rhythm). In AD, again only few researchers systematically compared the familiarity aspect, showing mixed results. Moussard et al. (2012, 2014) demonstrated a beneficial effect (only) on delayed recall of a sung presentation even when an unfamiliar melody was used, concluding that a sung presentation facilitates verbal memory regardless of the familiarity aspect. One study found evidence for improved recall after relearning the lyrics belonging to long-familiar songs as compared to lyrics belonging to a new song (Prickett & Moore, 1991). However, this could be due to reactivation of existing memory traces of previously learned lyrics, which is fundamentally different from learning new lyrics with a familiar melody.

To answer the question whether musical expertise leads to additional benefits of musical encoding, the findings indicate that musical expertise did not enhance beneficial effects of a sung presentation of information in most studies with cognitively unimpaired participants, except in one (Jellison, 1976). In AD studies, some researchers only included musically untrained participants while others did not systematically compare levels of musical expertise. However, Baird et al. (2017) included musical expertise as a covariate and demonstrated better learning of sung information in AD musicians compared to AD non-musicians.

### Underlying Mechanisms Proposed from Previous Studies

Several explanations have been provided for the positive results of music enhancing memory performance in cognitively unimpaired individuals and individuals with AD, related to automatic internal rehearsal (e.g., Calvert & Tart, 1993), enhanced structuring and chunking (e.g., Purnell-Webb & Speelman, 2008; Silverman, 2010, 2012), residual memory traces of familiar melodies (Baird & Samson, 2009), and emotional valence of the music (Ratovohery et al., 2018, 2019). These partly overlap with Ferreri and Verga’s (2016) model, in which a two-fold explanation focuses on the embedding of verbal material in musical structures on the one hand, and music-related effects of mood, arousal and reward on the other. In the following we will consider these ideas in the light of the reported findings.

#### Automatic Internal Rehearsal

Several authors put forward the notion that facilitation of delayed memory performance after musical embedding occurs because of automatic rehearsal of the music in the intermediate period (relative to a spoken presentation) (Calvert & Tart, 1993; Gfeller, 1983; Rainey & Larsen, 2002). Calvert and Tart (1993) refer to the experience of having a song stuck in your head, and the fact that one is thus automatically rehearsing the lyrics effortlessly. Reports from their participants revealed that they sang the words to themselves during a retrieval task. Calvert and Tart (1993) stated that repetition facilitates chunking the tune and words together (i.e., combining the accent structures of verbal and musical materials). Through this dual encoding, later retrieval efforts can be assisted by chunks of words that are stored with aid of the structural, repeating pattern of music. Researchers therefore concluded that songs are a helpful encoding, retrieval and recall strategy for long-term memory (e.g., Calvert & Tart, 1993; McElhinney & Annett, 1996).

#### Enhanced Structuring

Another explanation is that rather than repetition, the time structure or rhythm facilitates the ability to chunk (Purnell-Webb & Speelman, 2008; Silverman, 2010, 2012). Silverman (2007) concluded that rhythmic grouping resulted in pre-formed chunks that facilitated sequential recall and referred to past research on chunking into memory as a result of the use of rhythm (e.g., Schellenberg & Moore, 1985; Stoffer, 1985). However, in contrast to previous studies (e.g., Ee et al., 2015), Silverman et al. (2021) did not report significant differences between rhythm and no rhythm conditions. Purnell-Webb and Speelman (2008) concluded that a familiar rhythm, complementing the rhythm of the text, (with or without musical accompaniment) may provide the attachment of text to a schematic frame, thus possibly facilitating recall. Their findings were in line with the integration hypothesis as suggested by Serafine et al., (1984, 1986) who asserted that integrated in a melody, verbal material is changed and thus remembered differently. Both these ideas rest on the notion of a ‘joint accent structure’ created from the verbal material and the music, itself an integrated combination of the pattern of perceptual accents in pitch, rhythm and other kinds of musical structures (Jones, 1987), providing cues for memory by inducing enhanced attention to specific time points in the music. This mechanism is similar to what has been described in Dynamic Attending Theory (Jones, 1976; Jones & Boltz, 1989), which focuses on how attention is directed to specific points in temporally complex structures. Considering Purnell-Webb and Speelman’s (2008) findings, who referred to this joint structure as ‘prosodic match’, the dynamic attending mechanisms would direct attention to the structure resulting from integrating verbal material with a melody or rhythm. Thus, this may facilitate memory, especially if the accent structure of the melody and verbal material are well-matched. Ferreri and Verga (2016) also build their framework on the idea that melodic and rhythmic aspects of music provide a template contributing to the formation of internal rhythm in cortical networks involved in learning and memory. Notably, as the verbal material often also has a temporal structure of accents, this is merged with the accent structure in the music when verbal material is embedded, with varying levels of fit between the words and the music they are set to. It is likely that well-fitting accent structures lead to less complex stimuli, perhaps facilitating encoding. 

In AD it has been proposed that structuring mechanisms might also play a role (Moussard et al., 2012). Moussard et al. (2014) also referred to previous research supposing that the melody might provide cues to the structure of the lyrics (e.g., number of syllables per line) and limit the possibility of words to be set to the melody (i.e., Wallace, 1994).

#### Familiarity and Existing Memory Traces

The degree of familiarity of the melody (or rhythm) has also been proposed as a relevant aspect of music enhancing verbal memory and which is hypothesized to build on existing memory traces. Korenman and Peynircioglu (2004) used music snippets of varying instrumental and melodic familiarity and found enhanced recall in students when melodic familiarity increased. However, the downside of using a well-known melody may be that there is interference between the new verbal material to be learned and the previously overlearned lyrics belonging to a familiar tune. To avoid this potential problem, some authors specifically chose to use an unknown song (e.g., McElhinney & Annett, 1996) or to achieve familiarity with an unfamiliar melody prior to the actual experiment (e.g., Good et al., 2015; Oostendorp & Montel, 2014; Tamminen et al., 2017). Van den Bosch et al. (2013) showed that the level of expectation and predictability which is mediated by exposure to music, plays an important role in the arousal caused by the music. So, it could well be that using music that is to some degree familiar improves verbal memory through arousal.

In cognitively unimpaired older adults, some researchers have shown a beneficial effect of musical mnemonics (Moussard et al., 2014; Palisson et al., 2015; Ratovohery et al., 2018, 2019; Simmons-Stern et al., 2012); all of them used a familiar melody, except Simmons-Stern et al. (2012). Moussard et al. (2014) varied the degree of familiarity and found positive results of the highly familiar condition only in older adults. Ratovohery et al. (2019) supposed that in AD, a richer multimodal encoding may be the underlying mechanism of a familiar melody improving verbal memory. The previous results showed that aging individuals and individuals with (even severe) memory impairment can also benefit from musical mnemonics. Given the mixed results on familiarity it can be hypothesized that familiarity might be linked with arousal mechanisms, possibly improving verbal memory in cognitively unimpaired participants, whereas music in general—regardless of the familiarity aspect—may cause arousal and reward mechanisms more easily in AD, where cognitive resources may be less available.

#### Emotional Valence

Several authors note that music seems easier to retain than verbal material, sometimes interpreted to be due to the strong emotional power of music enhancing consolidation of memory traces (Ferreri & Verga, 2016; Samson et al., 2009).

Others revealed that specifically positively valenced music improved encoding in cognitively unimpaired older adults (Ratovohery et al., 2018), consistent with the positivity effect which has been frequently reported (e.g., Kalenzaga et al., 2016) in normal aging. Furthermore, it was found in AD that positively valenced music seemed to improve only immediate performance (Ratovohery et al., 2019).

However, it has also been reported that both positive and negatively valenced music improved delayed (10 min) verbal memory performance (Ratovohery et al., 2019). In line with this results, it is suggested that it is the musical experiences themselves, regardless of valence, that is generally more associated with positive emotions and memories in AD, leading to reward feelings, enhancing recollection.

### Explanations and Interpretation of Conflicting Results

On one side, studies in cognitively unimpaired young adults generally showed a positive effect of musical mnemonics, on the other side studies suggested that music decreases the memory performance through distraction and divided attention (Ferreri & Verga, 2016). In cognitively unimpaired older adults results were also mixed, and in AD we found a heterogeneity within the positive results. We will here briefly discuss the conflicting results and interpretations of these outcomes.

Various explanations for non-significant results in cognitively unimpaired participants have been given, relating to varying aspects, such as complexity of the verbal stimuli (e.g., unusual words, unconnected versus connected text), musical stimuli (e.g., (un)familiarity), personal aspects (e.g., musical expertise), task or practice specifics (e.g., presentation rate, rehearsal time, modality shift, memory paradigm), and stimulus complexity in relation to subsequent cognitive load and selective attention.

With respect to the complexity of the verbal stimulus, researchers, for example, reported the use of unusual words (Chazin & Neuschatz, 1990) or unconnected text instead of meaningful connected information (Rainey & Larsen, 2002). Moore et al. (2008) concluded in their study on persons with MS with regards to the musical stimulus, that the degree of familiarity with the used song was sometimes insufficient. Silverman (2007) concluded that the use of unfamiliar melodies may have resulted in working memory overload. Lehmann and Seufert (2018) suggested that the fact that the melody they used did not differ between every verse line, potentially could have led to simultaneous activation of multiple verse lines, consequently not being specific enough to function as an anchor. With regard to personal aspects, Rainey and Larsen (2002) hypothesized that differences in musical expertise (leading to differences in the degree of sensitivity to and effective use of musical elements, e.g., melody and rhythm) could play a role in the benefit of music as a prompt at initial learning. Regarding task specifics, Kilgour et al. (2000) thought that the success of a sung presentation might rely only on an artefact of presentation rate, which was also controlled for in other studies (e.g., Good et al., 2015; Ludke et al., 2014). Non-significant results can also be explained by insufficient rehearsal time, the memory paradigm used, or a modality shift between the training and testing phase (Moore et al., 2008). Interestingly, Good et al. (2015) indeed found that when participants were allowed to choose in the testing phase whether they wanted to speak or sing, the children who learned the information sung almost all chose to sing it back. Concerning stimulus complexity, Racette and Peretz (2007) supposed that singing is at least in the first steps of learning to perform a dual task, because of possible separate memory representations of text and melody.

In cognitively unimpaired older adults, several explanations have been provided for the mixed results of musical mnemonics. Most of the previous studies included them as controls to AD, which might have led to a ceiling effect (Ratovohery et al., 2018). Ratovohery et al. (2018) mentioned that the use of a recognition paradigm could have been too easy (e.g., Deason et al., 2012; Simmons-Stern et al., 2010, 2012).

Turning to the heterogeneity of the predominantly positive results in the AD population, Ratovohery et al. (2019) stated for example that the retention delay that was too long in relation to the disease severity could explain the absence of positive results in research of their colleagues (e.g., Baird et al., 2017). Moussard et al. (2012) concluded in their case study that singing at initial learning might not help memorization (or only when using a familiar melody). They referred to the theory of dual representation of song lyrics and the melody (cf. Hébert & Peretz, 2001; Peretz, 1996; Peretz et al., 2004), and hypothesized that this causes a slow and demanding initial memorization in AD but provides a robust trace, facilitating the retrieval from long-term memory (cf. Calvert & Tart, 1993; McElhinney & Annett, 1996; Rainey & Larsen, 2002; Wilson et al., 2006).

#### Model of Musical Mnemonics in Aging and AD

The aforementioned explanations for the beneficial effects of music as a mnemonic aid indicate that several factors must be taken into account: the complexity of the verbal stimulus (e.g., words, digits, sentences, stories), various aspects of the musical stimulus (e.g., simple or more complex rhythms of melodies, familiarity, emotional valence), together resulting in an overall stimulus complexity, and personal aspects (e.g., age, cognitive ability (cognitively unimpaired participants, cognitively unimpaired older adults, persons with MCI or AD), musical expertise, musical responsivity), in combination with task and practice specifics (e.g., presentation rate, repetition, level of participation, rehearsal time, modality congruence between training and testing, memory paradigm). The embedding of the verbal material in the musical stimulus possibly activates diverse mechanisms such as automatic internal rehearsal, enhanced structuring and chunking, richer multimodal encoding and the eliciting of emotion, arousal and reward mechanisms. We assume based on the diverging results of previous research, that in each individual a specific combination of these factors influences the degree of cognitive load, selective attention, and the affective response, resulting in an enhanced, unaffected, or even degraded performance in working memory encoding and long-term memory retrieval.

**Fig. 2:**
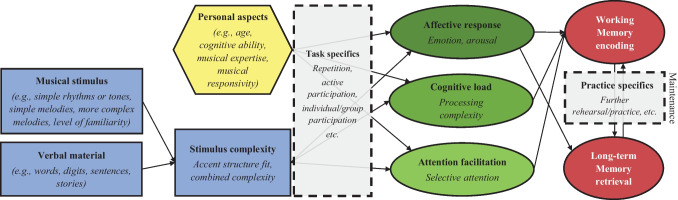
Proposed Model of the Effects of Musical Mnemonics on Memory Function. The model includes aspects of the verbal material (e.g., words, digits, sentences, stories), the music used for stimulus embedding (e.g., simple rhythms or tones, simple melodies, more complex melodies, level of familiarity), task specifics (e.g., repetition, level of participation), and personal aspects (e.g., age, cognitive ability, musical expertise, musical responsivity). Person-specific aspects are shown in a hexagon, cognitive processes are shown in ovals, external stimulus, task-, and practice specifics with boxes. The (mis-)match between accent structures of the musical stimulus and verbal material together contributes to the stimulus complexity (i.e., accent structure fit, or combined complexity). The latter influences the affective response, cognitive load, and attention facilitation, resulting in more or less effective working memory encoding. The affective response, cognitive load and attention facilitation are also dependent on personal factors such as age, age-related differences in emotion recognition, cognitive ability (i.e., cognitively unimpaired participants, cognitively unimpaired older adults, persons with MCI or AD) and musical expertise and responsivity. The two grey colored boxes with dotted lines represent task specifics (i.e., amount of repetition, modality congruence, memory paradigm, active participation) and practice specifics (i.e., rehearsal, more practice) that possibly play a role in working memory encoding, maintenance (e.g., further rehearsal, practice) and long-term memory retrieval

The above considerations can be summarized in a theoretical framework (see Fig. 2), thereby building on the sung vs. spoken part of the model by Ferreri and Verga (2016) as we hypothesize it applies to cognitively unimpaired older adults, and persons with MCI or AD. Specifically, we further elaborate on the nature of full stimulus complexity by including the result of the complexity of the verbal and musical stimulus separately and their accent structure fit, as well as personal and task characteristics, in the context of cognitive load (which may be especially relevant for aging or cognitively impaired populations).

The model indicates several ways in which musical embedding of verbal material may support memory functioning, and includes the aforementioned aspects that might play a role in making music a successful mnemonic aid in various points of the process of memorization, such as aspects of the verbal material, the musical stimulus embedding, task and practice specifics, and personal aspects (e.g., age, cognitive ability). The musical stimulus and verbal material together might create a match (or mismatch) resulting in the overall stimulus complexity for that specific pairing (based on complexity of each separate element, and accent structure fit). The combined accent structure thus represents the combination of the emphasis in the verbal material and the accents in the music and how well they match. The overall complexity is the outcome of this combination of accent structures; a good fit provides a more coherent and integrated accent structure and thus less complexity than when music and text do not fit very well. This complexity level influences the affective response (cf. North & Hargreaves, 1995), cognitive load, and attention facilitation, resulting in a more or less effective working memory encoding. The affective response, cognitive load and attention facilitation are also influenced by personal factors. Furthermore, task specifics not only potentially influence this affective response, cognitive load and attention facilitation, but possibly also affect working memory encoding, maintenance (e.g., further rehearsal, practice) and long-term memory retrieval (e.g., by eliciting an affective response; Ferreri & Verga, 2016).

Different elements in the model may be crucial to different populations, with the importance of each model element based on the characteristics of the population at hand. Specific mechanisms might be activated, for example, through positively valenced music in cognitively unimpaired older adults or through musical embedding in general in AD, causing activation of arousal, emotional and reward systems, possibly leading to enhanced memory performance. Although no studies on MCI were found in the current systematic review, this model may cover specific mechanisms for this population as well.

It is also important to note that our model extends, yet also differs from the one proposed by Ferreri and Verga (2016). While there are several similarities with the ‘sung versus spoken’-part of the framework put forward by Ferreri and Verga (2016), we here further elaborate the nature of overall stimulus complexity, which not only includes (1) the complexity of verbal or musical stimulus but notably also (2) the accent structure fit between verbal and musical stimulus and argue that there is a need for future studies to further clarify and test relationships between overall stimulus complexity and memory outcome. As the stimulus complexity might affect cognitive load and attention facilitation, this may be especially relevant for aging or cognitively impaired populations.

### Limitations

The results of our systematic review should be interpreted with caution due to the mixed outcomes of the studies identified. Few studies systematically assessed the potential of specific musical components or the role of musical expertise to facilitate memory. The inconsistencies in the methodological approaches cannot be easily interpreted; studies differed in the complexity of verbal information to be learned and remembered and musical stimulus embedding, the memory domain (i.e., working or episodic memory), and the memory process of interest (encoding, maintenance, retrieval). Previous research has mostly focused on cognitively unimpaired young adults and those findings cannot be generalized to patient groups. The few patient studies often have small sample sizes, often without appropriate controls, and the severity of cognitive impairments is not always well-defined. However, AD patient studies reflect evidence-based steps in this direction. Finally, there is a risk of publication bias in this field of research, compounded with methodological issues that can lead to false positive results (cf. Sala & Gobet, 2020).

### Recommendations for Future Research

The model formulated above may be of help to further systematize methodologies, drive research questions, and stimulate precise reporting of the verbal stimulus, musical stimulus embedding, personal aspects, and task and practice elements used. Based on the existing literature, we created reporting guidelines for research on musical mnemonics (See Box 2). The degree of participation at initial learning, the comparison between a self-created or imposed mnemonic (Moore et al., 2008), and the familiarity of the music (Rainey & Larsen, 2002) need to be investigated more thoroughly. Additionally, with neuro-imaging techniques and monitoring of psychophysiological arousal (Tamminen et al., 2017), we may deepen our knowledge of the mechanisms through which a musical presentation influences cognitive and brain functions and behavior (cf. Ferreri & Verga, 2016).

To our knowledge, the existing research on AD has focused on episodic memory functioning. Furthermore, there was a lack of studies in MCI. However, Rainey and Larsen (2002) suggested to examine also the role of working memory, hypothesizing that music can be best used to provide an additional structure for people with a limited working memory capacity (as can be the case in MCI and AD) to improve the ability to transfer information to episodic memory. Therefore, there is a need for future studies on effects of musical mnemonics on working (and episodic) memory functioning in persons with MCI and AD.

Finally, good measurement instruments need to be developed to allow for more systematic comparison of the degree of musical expertise since this is a probable moderating factor in the degree of benefit of a musical mnemonic. Several validated questionnaires are available that not only look at formal training, but also take musical engagement, exposure, or responsiveness into account (e.g., Chin & Rickard, 2012; Mas-Herrero et al., 2013; Müllensiefen et al., 2014).

Box 2 Suggested Reporting Guidelines for Research on Musical Mnemonics To better specify underlying mechanisms of musical mnemonics, future researchers are recommended to report precisely on the musical stimulus embedding and testing procedure, participant characteristics and musical and verbal stimuli used, specifically:

**Musical Stimulus Embedding and Testing Procedure:**

- Presentation paradigm (i.e., sung vs. spoken/rhythmically spoken, or other)
- Learning phase (*encoding*): social setting (individual vs. group), active (i.e., singing) or passive (i.e., listening) rehearsal conditions, live or recorded presentation, specific modality (auditory, visually, combined or other), imposed or self-created mnemonic
- Tasks specifics: e.g., number of repetitions, amount of rehearsal time, potential control for confounders (e.g., equation of duration of sung and spoken stimuli)
- Testing phase (*maintenance and retrieval*): if possible use standardized outcome measures (to promote the inclusion of the study results in future meta-analysis), specify memory measure (active immediate or (long-term) delayed recall, passive recognition, or both), duration of retention delay, modality (spoken, sung, written, multiple choice) and modality congruence between learning and testing (same or different from learning phase, choice/no choice), and practice specifics (e.g., amount of rehearsal, cues)

**Participant Characteristics**

- Demographic variables (e.g., age, cognitive ability, other clinical characteristics)
- Musical background (ideally using validated questionnaires)

**Musical and Verbal Stimulus Material**

- Music/Verbal: Materials used for memorization (i.e., level of complexity, tones vs. chords, words vs. text, etc.). If self-composed or created for the study: provide musical scores or text as supplementary materials
- Music/Verbal: Degree of familiarity (unfamiliar, familiar/familiarized)
- Music/Verbal: Potential pairing of semantics to acoustical characteristics of a tune
- Music: Whether valence, emotional pleasantness, or genre was accounted for
- Verbal: Potential relevance of verbal stimulus for daily living (for memory-impaired persons)
- Verbal: If relevant, serial position of important information

## Clinical Implications

Based on the above, we can conclude that musical mnemonics may be beneficial in AD and represent a low-cost strategy for improving recall of a limited amount of information in persons with mild to moderate (and even severe) AD (Oostendorp & Montel, 2014; Ratovohery et al., 2019). Future interventions should be designed personalizing the musical stimulus (e.g., genre, emotional valence, pleasantness, familiarity) to individual aspects (e.g., age, cognitive capacity, musical expertise, responsivity, and preferences), in order to maximize the potential of compensating for memory problems in everyday life of persons with MCI or AD.

Factors such as the relevance of the lyrics for daily living (Moussard et al., 2014), pairing the semantics to the acoustical characteristics of a tune (Moussard et al., 2014), enough rehearsal time to initially learn new information (Moore et al., 2008), the number of repetitions, the place of the important information at the beginning or end (Silverman & Schwartzberg, 2019), the degree of participation at encoding, familiarity of the music, and self-creating of a mnemonic (Moore et al., 2008) are all important to keep in mind when designing a musical mnemonic together with the patient. Interestingly, evidence from a word learning paradigm with background music rather than a sung presentation suggests that social aspects of the learning setting have an independent contribution to learning outcomes from musical aspects, suggesting that both are relevant to consider in clinical settings (Verga et al., 2015).

## Conclusion

We report an overall beneficial effect of musical mnemonics (i.e., sung presentation of verbal information at encoding), although results of individual studies are mixed. Building on existing theoretical work, we formulated a model of the cognitive processes activated by musical mnemonics depending on stimulus complexity and personal aspects of persons with and without cognitive impairment. Aspects that appear promising include familiarity with the musical material and musical expertise in the participants, which require more extensive investigation. Consequently, more systematic research is needed to identify which musical aspects, possible mechanisms, and mediating or moderating factors play a contributing role in the application of musical mnemonics in MCI and AD.

### Supplementary Information

Below is the link to the electronic supplementary material.

Supplementary file1 (DOCX 40 KB)

## Data Availability

Not applicable.

## Declarations

### Registration

This systematic review was not preregistered and no protocol was published.
