# Supplementary material for: Musical Mnemonics in Cognitively Unimpaired Individuals and Individuals with Alzheimer’s Dementia: A Systematic Review
Source: Neuropsychol Rev. 2023 Apr 14;34(2):455–77. doi: 10.1007/s11065-023-09585-4 (PMC11166747; doi:10.1007/s11065-023-09585-4)
Supplement: Supplementary file 1 — Supplementary file1 (DOCX 40 KB) [file 11065_2023_9585_MOESM1_ESM.docx]

| **Article** | **Musical stimulus embedding** | | | **Broader study results** |
| --- | --- | --- | --- | --- |
|  | **Learning phase** | | |  |
|  | Individually/group | Active/passive | Recording/live |  |
| Baird et al., 2017 | Individually | Passive | Recording | L: AD NMu sung < spoken |
|  |  |  |  | AD Mu > NMu |
|  |  |  |  | DR & Re: Mu OA = Mu AD |
| Calvert & Billingsley, 1998 | Exp. 1: Small groups | Passive & active | Recording | Exp. 1: No paradigm of interest > Rep French sung > spoken, Rep English sung = spoken |
|  | Exp. 2: Individually | Passive | Live | Exp. 2: Sung < prose |
| Calvert & Tart, 1993 | Small groups | Passive | Recording | Exp. 1: No paradigm of interest > naturalistic study |
|  |  |  |  | Exp. 2: STR + LTR: |
|  |  |  |  | SE: Sung = spoken |
|  |  |  |  | RE: Sung > spoken |
| Chazin & Neuschatz, 1990 | Individually | Passive & active | Recording | IR: Sung > spoken |
|  |  |  |  | DR: Sung = spoken |
| Deason et al., 2012 | Individually | Passive | Recording | Re: Sung = spoken |
| Gfeller, 1983 | Individually | Passive & active | Recording | Exp. 1: SR: Sung < spoken |
|  |  |  |  | Exp. 2: ER: Sung > spoken |
| Good et al., 2015 | Classroom | Passive & active | Live | IP: Sung > spoken |
|  |  |  |  | IT: Sung > spoken |
|  |  |  |  | IR: Sung > spoken |
|  |  |  |  | DR: Sung > spoken |
|  |  |  |  | DT: Sung = spoken |
| Jellison, 1976 | Individually | Passive | Recording | Sung > spoken |
|  |  |  |  | Sung: ME > NME |
| Jellison & Miller, 1982 | Individually | Passive | Recording | Sung < spoken digit span |
|  |  |  |  | Sung = spoken word span |
|  |  |  |  | OR: ME = NME |
|  |  |  |  | ME > NME SeR verbal material |
| Kilgour et al., 2000 | Individually | Passive | Recording | Exp. 1: sung + sung with piano prelude > spoken (IR & DR) |
|  |  |  |  | Exp. 2 & 3: sung < spoken |
|  |  |  |  | ME>NME verbal material |
| Lehmann & Seufert, 2018 | Classroom | Passive | Recording | R: Visual > sung  R: Visual > spoken  R: Sung = spoken (‘only differed on a descriptive level…’)  C: Sung > visual  C: Spoken = visual  C: Sung = spoken |
| Ludke et al., 2014 | Individually | Passive & active | Recording | Sung > spoken Hungarian (IR & DR) |
| Ma et al., 2020 | Individually | Passive | Recording | WL: Sung & IDS > ADS |
|  |  |  |  | Delayed Recall: Sung & IDS > ADS |
|  |  |  |  | UF = F |
| McElhinney & Annett, 1996 | Small groups | Passive | Recording | 1 x: Sung = spoken |
|  |  |  |  | 2 & 3 x: Sung > spoken |
| Moussard et al., 2012 | Individually | Passive & active | Recording | IL: Sung UF < spoken |
|  |  |  |  | IL: Sung HF & LF > Sung UF |
|  |  |  |  | RL UF + F: Sung > spoken |
| Moussard et al., 2014 | Individually | Passive & active | Recording | IR: Sung = spoken |
|  |  |  |  | DR: Sung > spoken, OA: F, AD: F & UF |
| Oostendorp & Montel, 2014 | N.R. | Active | Live | FR + CR: Sung > spoken |
| Palisson et al., 2015 | Individually | Passive & active | Recording | IR: Sung > spoken & SME |
|  |  |  |  | DR: Sung > spoken & SME |
| Prickett & Moore, 1991 | Individually | Active | Live | WR: F song > new song/ F or new spoken |
|  |  |  |  | WR: F m% = 71.8 vs. new m% = 42.19 |
| Purnell-Webb & Speelman, 2008 | Individually | Passive | Recording | Exp. 1: R: Rhy (UF/F) > spoken & UF melody |
|  |  |  |  | Exp. 1: Rhy (UF/F) = Melody (F) |
| Racette & Peretz, 2007 | Individually | Passive & active | Recording | Exp. 1: IR & DR: Spoken = Sung  Exp. 2: No paradigm of interest |
| Rainey & Larsen, 2002 | Individually | Passive | Recording | Exp. 1 & 2: IL Sung = spoken |
|  |  |  |  | Exp. 1 & 2: RL Sung > spoken |
| Ratovohery et al., 2018 | Individually | Passive & active | Recording | EM YA > OA |
|  |  |  |  | OA IR + DR: Sung PV > spoken |
| Ratovohery et al., 2019 | Individually | Passive & active | Recording | OA > AD |
|  |  |  |  | AD Encoding + IR + DR (10 min & 24 hour): Sung > spoken |
|  |  |  |  | AD encoding: PV > NV |
|  |  |  |  | OA R: PV > NV |
| Rukholm et al., 2018 | Classroom | Passive | Recording | Productive & receptive learning:  Sung/HE > Sung/ LE, P/HE & P/LE |
| Schön et al., 2008 | Individually | Passive | Recording | Exp. 2 (sung constant syllable-pitch mapping > Exp. 1 (spoken) |
|  |  |  |  | Exp. 3 (sung variable syllable-pitch mapping ) > Exp. 1 (spoken) |
|  |  |  |  | Exp. 3 (sung variable syllable-pitch mapping) < Exp. 2 (sung constant syllable-pitch mapping) |
| Silverman, 2007 | Individually | Passive | Recording | Rhy > spoken |
|  |  |  |  | ME > NME verbal material |
| Silverman, 2010 | Individually | Passive | Recording | Rhy + |
|  |  |  |  | F - |
|  |  |  |  | ME > NME verbal material |
| Silverman, 2012 | Individually | Passive | Recording | Rhy > NRhy |
|  |  |  |  | ME = NME |
| Silverman & Schwartzberg, 2014 | Individually | Passive | Recording | M > Fe voice |
|  |  |  |  | Piano, N Acc > guitar |
|  |  |  |  | ME = NME |
| Silverman & Schwartzberg, 2019 | Individually | Passive | Recording | Au > V + Au |
|  |  |  |  | Au: Melody = sung = spoken |
|  |  |  |  | V + Au: Sung > spoken, Melody > spoken, Sung = Melody |
|  |  |  |  | ME = NME |
| Simmons-Stern et al., 2010 | Individually | Passive | Recording | AD Re Sung > spoken |
| Simmons-Stern et al., 2012 | Individually | Passive | Recording | GC Sung > spoken: OA & AD |
|  |  |  |  | SC Sung = spoken |
|  |  |  |  | FA: Sung > spoken: AD |
| Tamminen et al., 2017 | Individually | Passive | Recording | R: Sung = spoken |
|  |  |  |  | Re: Sung = spoken |
|  |  |  |  | IML: Sung F > spoken |
| Wallace, 1994 | Individually | Passive | Recording | Exp. 1: 3 vs Sung (OM) > spoken |
|  |  |  |  | Exp. 2: 3 vs Sung (OM) > rhythmic spoken |
|  |  |  |  | Exp. 3: 1 vs Sung < spoken |
|  |  |  |  | Exp. 4: 3 vs Sung (OM) > 3 vs sung (DM) or spoken |
| Wolfe & Hom, 1993 | Individually | Passive | Live | L: Sung F > UF & spoken |
|  |  |  |  | IR: Sung = spoken |
|  |  |  |  | Ret: Sung = spoken |
| Yalch, 1991 | Exp. 1: Classroom | Passive | Exp. 1: No paradigm of interest: List of slogans (jingle/no jingle format) | Exp. 1: Aided Recall: Jingle > No Jingle |
|  |  |  |  | Exp. 1: Passive Recognition: Jingle = No Jingle |
|  | Exp. 2: Small groups |  | Exp. 2: Recording | Exp. 2: Aided Recall: Jingle > No Jingle |
|  |  |  |  | Exp. 2: Passive Recognition: Jingle > No Jingle |
|  |  |  |  | Exp. 2: Interaction: Jingle Aided Recall > Jingle Passive Recognition |
|  |  |  |  | Exp. 2: Interaction: Jingle Aided Recall One Exposure > Two exposures |

*Note*: articles listed in alphabetical order. ‘Passive’ in the column learning phase means listening, when an active rehearsal condition was used, this is specified. If the effect concerned both groups this is not specified, if an effect concerned one of the groups this is separately mentioned in the results column. Abbreviations in alphabetical order: Acc = accompaniment; AD = Alzheimer’s dementia; ADS = adult directed speech; Au = auditory; C = comprehension; CR = cued recall; DM= different melodies; DR = delayed recall; DT = delayed translation; EM = episodic memory; ER = extended rehearsal; Exp. = experiment; F = familiar; FA = likely for false alarm: ‘to incorrectly identify a novel stimulus as ‘‘old’’’; Fe = female; FR = free recall; GC = general content; HE = high elaboration; IDS = infant directed speech; IL = initial learning; IML = integration mental lexicon; IP = immediate pronunciation; IR = immediate recall; IT = immediate translation; LE = low elaboration; LTR = long term recall; M = male; ME = musical expertise; Mu = musicians; NME = no musical expertise; NMu = non-musicians; NV = negative valence; OA = (cognitively unimpaired) older adults; OM = one melody; PV = positive valence; R = recall; Re = recognition; RE = repeated exposure; Rep = repetition; Ret = retention; Rhy = rhythm; RL = relearning; SC = specific content; SE = single exposure; SeR = sequential recall; SME = silent movie excerpts; SR = single rehearsal; STR = short term recall; UF = unfamiliar; V = visual; vs = verses; vs. = versus; WL = word learning; WR = word recall; YA = (cognitively unimpaired) young adults
